# Pooled Analysis of Risk Stratification of Spontaneous Type 1 Brugada ECG: Focus on the Influence of Gender and EPS

**DOI:** 10.3389/fphys.2018.01951

**Published:** 2019-01-31

**Authors:** Xinye Li, Frédéric Sacher, Kengo F. Kusano, Hector Barajas-Martinez, Nian Liu, Yanda Li, Yonghong Gao, Tong Liu, Hongcai Shang, Charles Antzelevitch, Dan Hu, Yanwei Xing

**Affiliations:** ^1^Guang'anmen Hospital, Chinese Academy of Chinese Medical Sciences, Beijing, China; ^2^College of Acupuncture and Massage, Beijing University of Chinese Medicine, Beijing, China; ^3^Hôpital Cardiologique Haut Lévêque, Lyric Institute, Université de Bordeaux, Bordeaux-Pessac, France; ^4^Division of Arrhythmia and Electrophysiology, Department of Cardiovascular Medicine, National Cerebral and Cardiovascular Center, Osaka, Japan; ^5^Lankenau Institute for Medical Research, Wynnewood, PA, United States; ^6^Beijing Anzhen Hospital, Capital Medical University, Beijing, China; ^7^The Key Laboratory of Chinese Internal Medicine of the Ministry of Education, Dongzhimen Hospital Affiliated to Beijing University of Chinese Medicine, Beijing, China; ^8^Tianjin Key Laboratory of Ionic-Molecular Function of Cardiovascular Disease, Department of Cardiology, Tianjin Institute of Cardiology, Second Hospital of Tianjin Medical University, Tianjin, China; ^9^Lankenau Institute for Medical Research and Jefferson Medical College, Philadelphia, PA, United States; ^10^Department of Cardiology and Cardiovascular Research Institute, Renmin Hospital of Wuhan University, Wuhan, China; ^11^Hubei Key Laboratory of Cardiology, Wuhan, China

**Keywords:** Brugada syndrome, risk stratification, gender, syncope, electrophysiology

## Abstract

**Aims:** Risk stratification of patients with Brugada syndrome (BrS) is vital for accurate prognosis and therapeutic decisions. Spontaneous Type 1 ST segment elevation is generally considered to be an independent risk factor for arrhythmic events. Other risk factors include gender, syncope, sudden cardiac arrest (SCA), and positive electrophysiological study (EPS). However, the further risk stratification of spontaneous type 1 combined with the other risk factors remains unclear. The present study pooled data from 4 large trials aiming to systematically evaluate the risk of spontaneous Type-1 ECG when combined with one or more of these other recognized risk factors.

**Methods:** We searched for related studies published from November 2, 2002 to February 10, 2018 in PubMed, EMBASE, Cochrane Library, MEDLINE, Chinese National Knowledge Infrastructure (CNKI), and Wanfang Databases. The pooled data were evaluated combining each risk factor with the presence of a spontaneous Type-1 ECG. All analyses were performed using Review Manager, version 5.0.12.

**Results:** Four eligible studies involving 1,338 patients (85% males, mean age: 48.1 ± 18.1 years) were enrolled. Spontaneous Type-1 ECG was associated with higher risk for ventricular tachycardia/fibrillation (VT/VF) than cases with non-Type 1 ECG in males (odds ratio: 95% CI: 1.84–5.17; *P* < 0.0001), but not in females (*P* = 0.29). Among spontaneous Type-1 cases with syncope or with positive EPS, the difference was not statistically significant (*P* = 0.06 and 0.07, respectively). Patients with Type-1 ECGs and positive EPS were at higher risk than those with negative EPS (95% CI: 1.10–5.04; *P* = 0.03). Pooled analysis showed an association of Spontaneous Type-1 ECG, Type-1 ECGs combined with male, and Type-1 ECGs combined with positive EPS between increased risk of arrhythmic events.

**Conclusion:** Our results indicate that in BrS patients, a spontaneous Type-1 ECG is an independent risk factor for SCD in males, but not in females. A spontaneous Type-1 BrS is associated with a worse prognosis when combined with positive EPS

## What's New?

- Original data are collected to perform a pooled analysis, providing a systematic evaluation assessing the risk of spontaneous Type 1 BrS combined with gender, syncope, SCA, and EPS.
- In BrS patients, a spontaneous Type-1 ECG is an independent risk factor for SCD in males, but not in females.
- The prognosis of spontaneous Type 1 BrS patients is worse when combined with positive results of EPS.

## Introduction

Brugada syndrome (BrS) is a rare heritable arrhythmia syndrome characterized by the presence of ST-segment elevation in the right precordial leads (V1/V2) in the electrocardiogram (ECG) (Benito et al., 2008a; Gütter et al., 2013). It is associated with sudden cardiac death (SCD) due to ventricular tachycardia/fibrillation (VT/VF) (Letsas et al., 2011). BrS is diagnosed in patients with a coved-type ST-segment elevation (Type 1) ≥2 mm in ≥1 right precordial leads positioned in the 2nd, 3rd, or 4th intercostal space, occurring either spontaneously or after a provocative drug test involving intravenous administration of potent sodium channel blockers (ajmaline, flecainide, pilsicainide, or procainamide) (Priori et al., 2013). Recently, the new consensus report suggests that when a Type 1 ST segment elevation is exposed using sodium channel blockers, the diagnosis of BrS should require that the patient also present one of the following cases: syncope of probable arrhythmic cause, a recorded VF or polymorphic ventricular tachycardia, a family history of SCD in the age of <45 with negative autopsy, nocturnal agonal respiration, or coved-type ECGs in family members (Antzelevitch et al., 2016).

A spontaneous Type 1 ECG is reported to be an independent predictor of VF (Priori et al., 2012). Spontaneous type 1 pattern in the precordial leads were associated with later cardiac events (Tokioka et al., 2014). Several clinical factors have been shown to be associated with a worse outcome in patients with BrS (Priori et al., 2013), and it was shown that SCN5A mutation might be possible to improve the risk stratification systems for BrS (Li et al., 2018). The majority of studies have provided evidence that patients with a spontaneous Type 1 ECG at baseline are at relatively high risk for cardiac arrhythmic events during the follow-up period, particularly if they have a history of syncope (Benito et al., 2008b; Kamakura et al., 2009; Priori et al., 2012, 2013). Appropriate treatment is not significant in asymptomatic BrS patients (Sacher et al., 2013). When diagnosed, Type 1 ECG was significantly more common in men than in women (Benito et al., 2008b). Recently a study indicated that female patients with BrS were much rarer, displayed less Type 1 Brugada ECG and had a lower induction rate than males (Milman et al., 2018b). Also, the risk of arrhythmia events in male patients was higher than that in female patients. Meanwhile, in the male population, symptomatic patients had significantly higher risk than asymptomatic patients (Yuan et al., 2018). Type 1 ECG is associated with greater risk when compared to the non-Type 1 Brugada pattern, but no studies have provided a systematic evaluation assessing the risk of Type 1 BrS combined with gender, syncope, sudden cardiac arrest (SCA), and electrophysiological study (EPS).

Our aim in this study is to perform a pooled analysis of available data from patients diagnosed with BrS so as to assess the prognostic significance of spontaneous Type 1 ECG as a function of gender and in association with other risk factors including syncope and positive EPS.

## Methods

We report our systematic review and meta-analysis according to the statement for meta-analyses of Preferred Reporting Items for Systematic reviews and Meta-Analyses (PRISMA) (Hutton et al., 2015).

### Search Strategy and Inclusion Criteria

Two investigators independently and comprehensively divided the work to perform a literature search. One investigator performed the review of PubMed, EMBASE, and the Chinese National Knowledge Infrastructure (CNKI) database to find relevant researches. Another investigator identified relevant studies by performing a literature search of the Cochrane Library, MEDLINE, and Wanfang Databases. In order to identify and retrieve all potentially relevant articles regarding this topic, we searched the related studies published from November 2, 2002 to February 10, 2018 utilizing the following query terms: “Brugada” and “syndrome,” or “Brugada syndrome” and “Type 1,” or “risk stratification.” In addition, the titles, abstracts, and reference lists of all articles were carefully reviewed. In addition, reference lists of published articles were searched for additional publications. The relevant studies were retrieved as full text and assessed for compliance with the inclusion criteria by two investigators. In the case of multiple reports from the same group of authors, the one containing the largest number of patients was selected in order to prevent duplication of data. Studies were considered for this pooled analysis only if they were full-size articles in written in English and published in peer reviewed journals. Our inclusion criteria were as follows:

- the study design was a prospective or retrospective observational study;
- patients with a spontaneous Type 1 ECG BrS pattern or non-Type 1 ECG BrS pattern;
- a follow-up duration of ≥ 10 months to permit detection of arrhythmic events;
- contained information on clearly defined endpoints (appropriate ICD shocks, VF/VT, and SCD);
- studies with full-text;
- risk ratio (RR), hazard ratio (HR), odds ratio (OR), corresponding 95% confidence intervals (CIs), or necessary raw data were reported.

Studies reporting only composite endpoints but no particular data on all-cause mortality or dealing with distinct patient populations were not considered. To resolve the disagreements and uncertainties between the two investigators, a consensus had been reached after reviewing the source data or consultation with a third investigator.

We sent e-mails to the principal authors of identified studies to ask for data sharing using a standardized form and definitions. Then the original data of these articles were collected. The centers were requested to state institutional Review Board approval and informed consent in order to avoid any ethical issues. Additional patients evaluated during the follow-up period after publication of the studies were included. As a consequence, the patient number reported here might be different from that published in the original reports.

### Data Extraction

The extracted data elements of this pooled analysis consisted of: surname of first author, publication year, origin of the studied population, type of study, study design, sample size, participants' age and gender, duration of follow-up, end-point events, the quality score, number of subjects with spontaneous Type 1 ECG pattern, number of subjects with family history of SCD or syncope, positive SCN5A gene mutation, detailed information in relation to programmed ventricular stimulation (PVS), positive number of inducible VT/VF, and the presence of a fragmented QRS.

### Quality Assessment

All studies included in our pooled analysis underwent quality assessment using the Methodological Index for Non-Randomized Studies (MINORS) (Slim et al., 2003) (**Table 2**). A point score system in which each item is scored from 0 to 2 with a maximum score of 24 points was used for the following characteristics: aim of the study, inclusion of consecutive patients, prospective collection of data, appropriate endpoint to the study aim, unbiased evaluation of endpoints, follow-up period appropriate to the chief endpoints, loss to follow-up not exceeding 5%, comparable control group provided with the gold standard interventions, contemporary groups, baseline equivalence of groups, prospective calculation of the sample size, adequate statistical analysis using in the study design. Both reviewers independently scored the selected publications, and then used the average MINORS score for final assessment. According to their MINORS scores of <16 and ≥16 points, studies were defined to be low-quality and high-quality studies separately. The standard deviation of mean quality score was 16.5 ± 1.4.

### Statistical Analysis

Results of the cardiac events outcome are expressed as odds ratios (ORs) with 95% confidence intervals (CIs) for each study. *I*^2^ derived from the standard chi-square test, which represented the percentage of the variability in effect estimates produced by heterogeneity, was used rather than sampling error to evaluate heterogeneity across studies. An *I*^2^ > 50% was symbolic of significant statistical heterogeneity (Higgins et al., 2003). In this case, the random-effects model using the inverse variance heterogeneity method was used to consider within-study and between-study variance. Otherwise, the pooled effect was calculated with a fixed-effects model.

In sensitivity analyses, after eliminating any one of the articles, neither *I*^2^ nor *P*-values had changed. Subgroup analyses were performed based on whether incorporative effect sizes were adjusted in the BrS patients. Besides, we used a random predefined manner to perform the sensitivity analysis. Publication bias was assessed by means of the funnel plot. Statistical significance was determined a definition at *P* ≤ 0.05. All analyses were performed using Review Manager, version 5.0.12 (Revman; The Cochrane Collaboration, Oxford, UK).

## Results

### Study Selection

**Figure 1:**
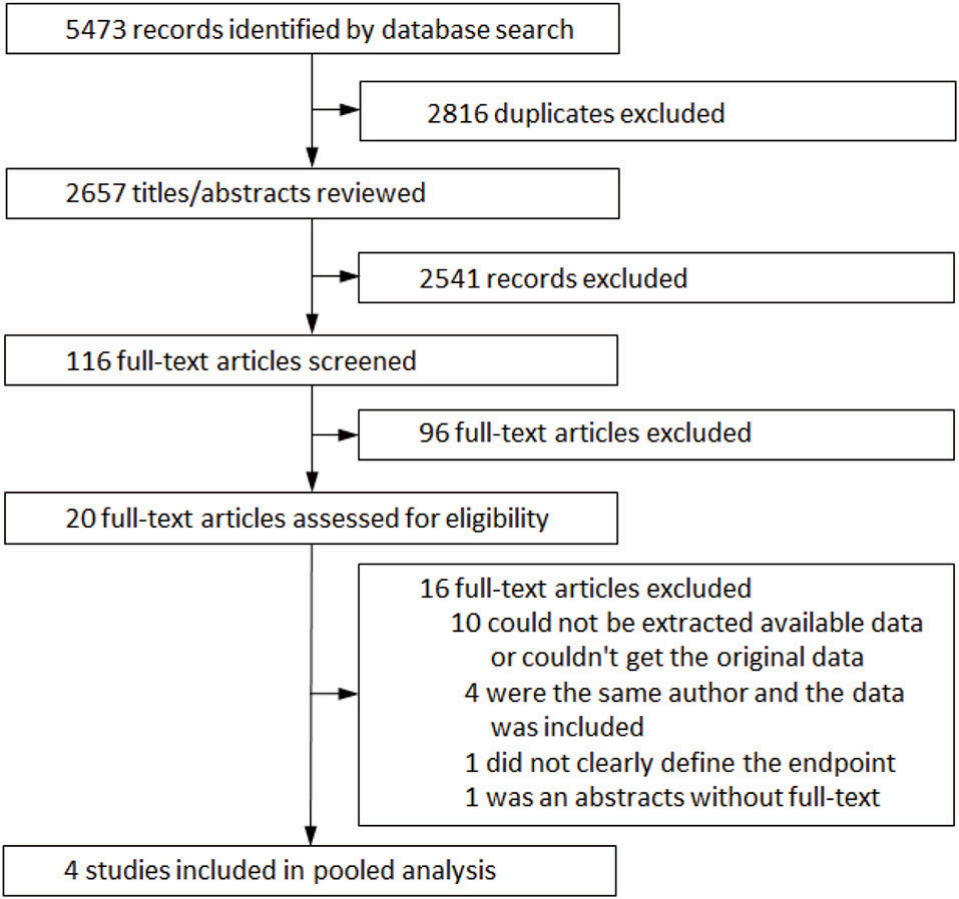
Flow diagram showing parameters for data search and study selection.

Figure 1 illustrates a flow diagram of the data search and study selection. Using our search criteria, the systematic review of the literature provided a total of 5,473 potentially relevant studies. After screening the titles and abstracts, 2,541 studies were rejected. Accordingly, 20 potentially correlative studies were searched for detailed evaluation. At last, 4 studies (Benito et al., 2008b; Kamakura et al., 2009; Sacher et al., 2013; Tokioka et al., 2014) met the pre-defined systematic review search criteria and were incorporated into our analysis.

Twenty studies consisted of 4,675 BrS patients in total (Supplemental Tables 1, 2). A spontaneous Type 1 ECG pattern of BrS was reported in 2,503 patients. Four studies were eligible for this pooled analysis consisting of 1,338 patients (85% males, mean age: 48.1 ± 18.1 years) with BrS in total. Among these were one single-center and three multicenter studies and meanwhile two prospective and two retrospective studies (Table 1). Table 2 summarized the patients' general characteristics included in each study. The average follow-up period ranged from 10 to 119 months. The definition of Type 1 Brugada-pattern ECG was similar in all studies. A spontaneous Type 1 ECG pattern of BrS was reported in 709 patients. As for subgroups analysis: 100 had survived an episode of SCA, 289 with a history of syncope and 373 were asymptomatic. In addition, 1,048 (78%) patients underwent PVS. In all four studies, electrophysiology study involved up to three extra-stimuli. The rate of inducibility of ventricular arrhythmias was 40% (*n* = 416).

**Table 1:** Characteristics of the four studies enrolled in the pooled analysis.

**Investigator**	**Location**	**Type of study**	**Study design**	**Study population**	**Mean follow-up**	**Endpoint**	**Quality score**	**Mean±SD of quality score**
Benito et al., 2008b	Canada	MC	PS	Patients with spontaneous or sodium-blocker induced Type 1 coved-type ECG pattern (Type-1 ECG)	58 ± 48 months	SCD/Documented ventricular fibrillation	15	16.5 ± 1.4
Kamakura et al., 2009	Japan	MC	RS	Patients with a Type 1 or non-Type 1 Brugada ECG pattern	48.7 ± 15.0 months	VF/Sudden death	18	
Sacher et al., 2013	America	MC	PS	Patients with a Type 1 Brugada ECG pattern implanted with an ICD	77 ± 42 months	Death/Inappropriate shock	16	
Tokioka et al., 2014	Japan	SC	RS	Patients with a Brugada-Type 1 ECG	45.1 months	VF/SCD	17	

*ECG, electrocardiogram; ICD, implantable cardioverter defibrillator; MC, multicenter study; MINOR, methodological index for non-randomized studies; PS, prospective study; RS, retrospective study; SC, single center study; SCD, sudden cardiac death; VF, ventricular fibrillation; SD, standard deviation*.

**Table 2:** Clinical characteristics of patients included in the study.

	**Benito et al., 2008b**	**Kamakura et al., 2009**	**Sacher et al., 2013**	**Tokioka et al., 2014**
Total patients, *n*	384	330	378	246
Male/female, *n*	272/112	315/15	310/68	236/10
Age (years)	48 ± 18	51 ± 15	46 ± 13	48 ± 14
Spontaneous Brugada ECG, *n* (%)	154 (40)	173 (52)	226 (60)	156 (63)
Non-spontaneous Type 1 Brugada ECG, *n* (%)	230 (60)	157 (48)	152 (40)	90 (37)
Family history of SCD, *n* (%)	NA	30 (9)	111 (29)	69 (28)
History of syncope, *n* (%)	65 (17)	67 (20)	181 (48)	40 (16)
Spontaneous Type 1 with Syncope total, *n* (%)	NA	46 (14)	107 (28)	28 (11)
Spontaneous Type 1 with syncope events, *n* (%)	NA	1 (0)	18 (5)	12 (5)
Asymptomatic spontaneous Type 1 total, *n* (%)	103 (27)	154 (47)	93 (25)	NA
*SCN5A* mutation, *n* (%)	95 (25)	NA	41 (11)	17 (7)
**EPS**				
Stimulation sites	RVA	RVA+RVOT	NA	RVA+RVOT+LV
Extra stimuli	Up to 3	Up to 3	Up to 3	Up to 3
Basic cycle lengths	600, 500, and 430 ms	NA	NA	2 cycles
Patients with EPS, *n* (%)	350 (91)	232 (70)	311 (82)	155 (63)
Inducible VT/VF, *n* (%)	95 (27)	22 (9)	228 (73)	71 (46)
fQRS (+), *n* (%)	NA	NA	NA	78 (32)

*ECG, electrocardiogram; EPS, electrophysiological study; fQRS, fragmented QRS; LV, left ventricle; NA, not available; n, number; RVA, right ventricular apex; RVOT, right ventricular outflow tract; SCD, sudden cardiac death; VF, ventricular fibrillation; VT, ventricular tachycardia*.

### Type 1 and Non-type 1 Groups

Overall, Type 1 BrS patients displayed an increased risk of arrhythmic events compared to non-Type 1 BrS patients (OR 2.88, 95% CI: 2.28 to 3.63, *P* < 0.00001; Heterogeneity: *P* = 0.01, *I*^2^ = 46%, Supplemental Figure 1). The calculations showed a statistically significant difference between the two groups. We conducted sensitivity analysis, excluding any data sets that would have no effect on the results.

### Gender Groups

**Figure 2:**
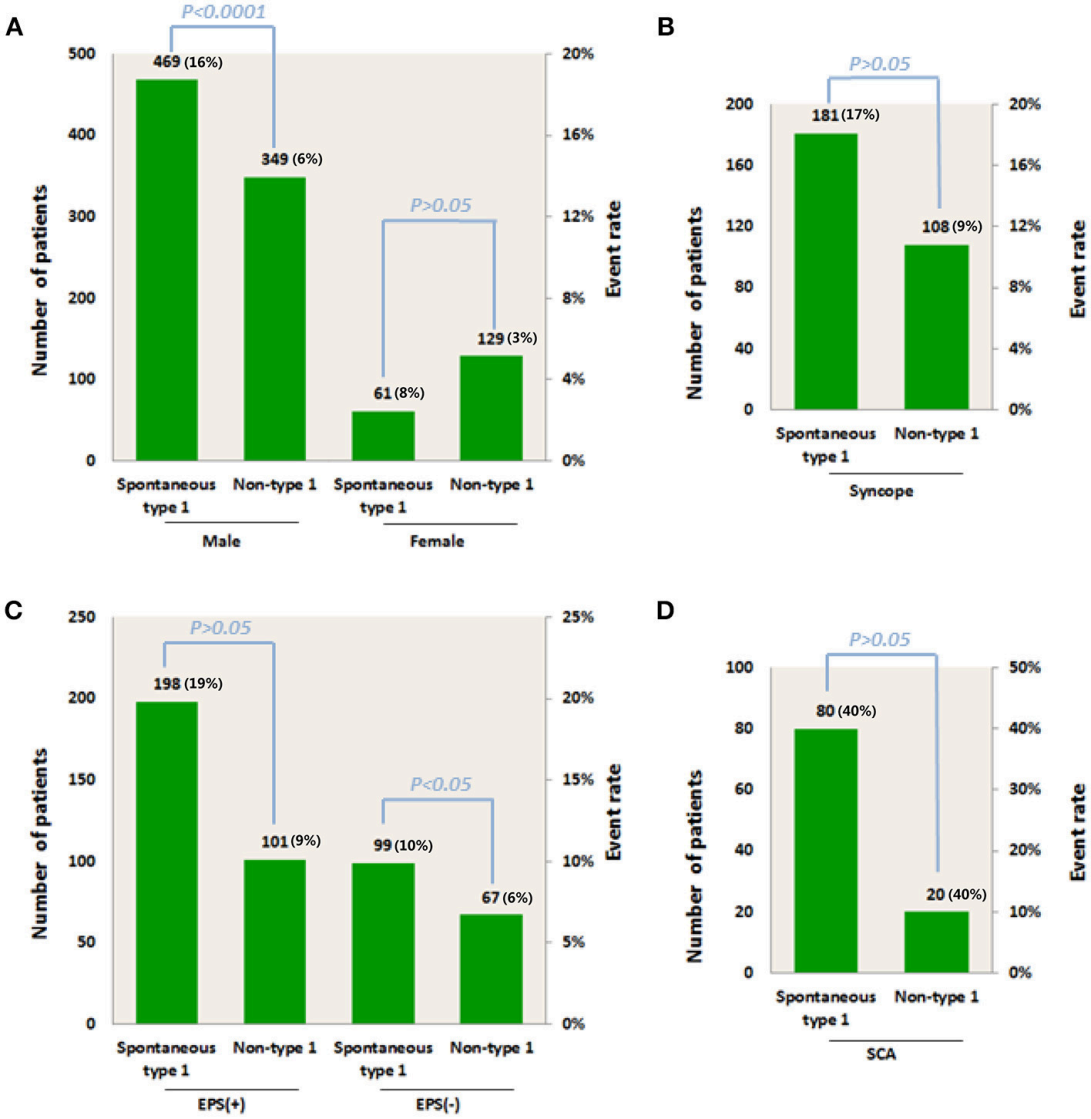
Total number of patients and arrhythmic event rate for patients with spontaneous Type 1 or non-Type 1 ECGs in association with **(A)** Male or female gender group; **(B)** Syncope; **(C)** Positive or negative EPS, and **(D)** SCA. EPS, electrophysiological study; SCA, sudden cardiac arrest.

**Figure 3:**
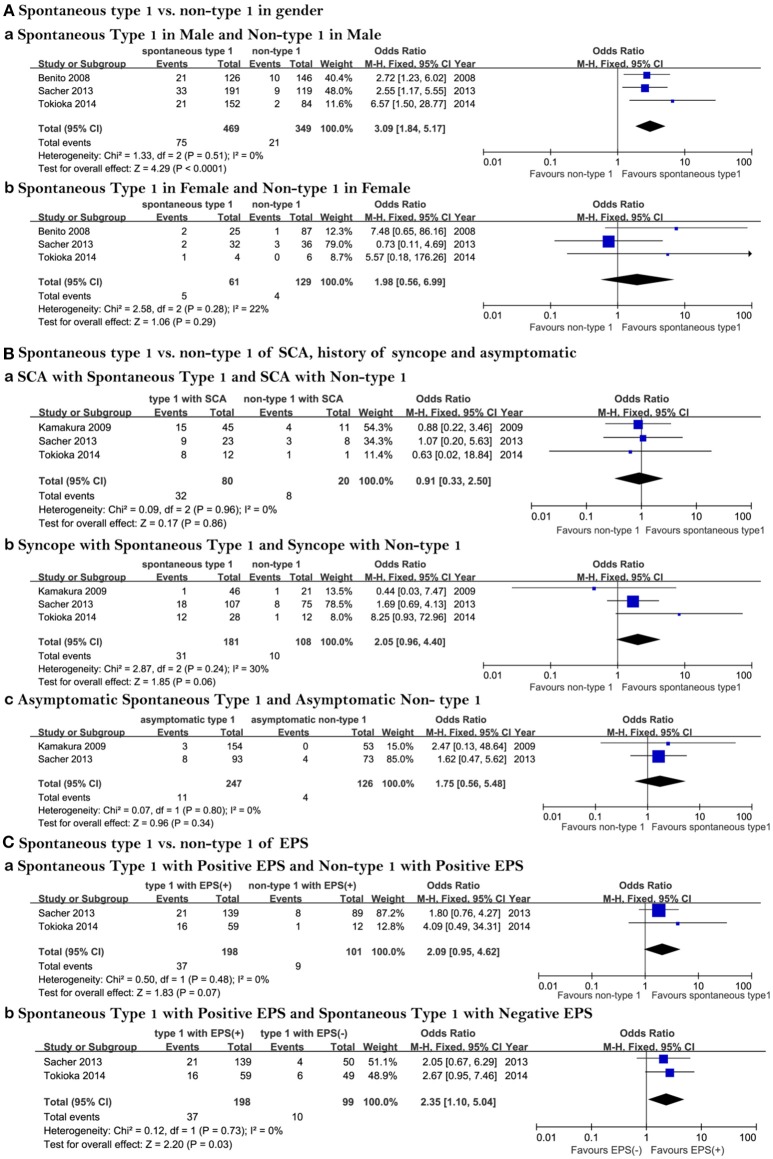
Forest plots comparing outcomes of subgroups. **(A)** Spontaneous Type 1 vs. non-Type 1 as a function of gender, (a) Prognosis of spontaneous Type 1 and non-Type 1 in males, (b) Prognosis of spontaneous Type 1 and non-Type 1 in females; **(B)** Spontaneous Type 1 vs. non-Type 1 in patients experiencing SCA, history of syncope and asymptomatic patients, (a) Prognosis of spontaneous Type 1 SCA and non-Type 1 in patients experiencing aborted SCA, (b) Prognosis of spontaneous Type 1 and non-Type 1 in patients with a history of syncope, (c) Prognosis of spontaneous Type 1 and non-Type 1 in asymptomatic patients; **(C)** Spontaneous Type 1 vs. non-Type 1 in patients with positive and negative EPS, (a) Prognosis of spontaneous Type 1 and non-Type 1 ECG in patients with positive EPS and non-Type 1 with positive EPS, (b) Prognosis of spontaneous Type 1 and non-Type 1 ECG in patients with negative EPS. EPS, electrophysiological study; SCA, sudden cardiac arrest.

Three articles were included in which we could extract the data we need from raw data (Benito et al., 2008b; Sacher et al., 2013; Tokioka et al., 2014). Of the 530 individuals with a Type 1 ECG, 469 (88%) were male. Of the 478 Individuals without a Type 1 ECG, 349 (73%) were male. The event rate in male patients with spontaneous Type 1 ECG was higher than that of Types 2 and 3 (16 and 6%, respectively). In females, the corresponding incidence was 8 and 3% (Figure 2A). Male patients who developed spontaneous Type 1 Brugada pattern were at higher risk than patients with non-Type 1 BrS (OR 3.09, 95% CI: 1.84 to 5.17, *P* < 0.0001; Heterogeneity: *P* = 0.51, *I*^2^ = 0%, Figure 3Aa). Interestingly, the risk for women did not significantly differ between spontaneous Type 1 and non-Type 1 (OR 1.98, 95% CI: 0.56 to 6.99, *P* = 0.29; Heterogeneity: *P* = 0.28, *I*^2^ = 22%, Figure 3Ab). The presence or absence of a spontaneous Type 1 Brugada pattern was not significantly associated with risk for future events in female patients.

### SCA, Syncope, and Asymptomatic Groups

A total of 762 BrS patients were included. Information of asymptomatic individuals and patients with syncope or SCA were provided in three studies (Kamakura et al., 2009; Sacher et al., 2013; Tokioka et al., 2014). The proportion of spontaneous Type 1 and non-Type 1 of the three papers is: 63 vs. 37%; 80 vs. 20%; 66 vs. 34%, respectively. Of all three groups, there was not a high risk of events among those patients with spontaneous Type-1 ECG. The rate of arrhythmic events was similar (40%, Figure 2D) whether SCA was combined with spontaneous Type 1 and or Type 2 or 3 (40%, Figure 2D). Arrhythmic events were no different between Type 1 and non-Type 1 BrS patients presenting with SCA (OR 0.91, 95% CI: 0.33 to 2.50, *P* = 0.86; Heterogeneity: *P* = 0.96, *I*^2^ = 0%, Figure 3Ba). The corresponding incidence in the syncope group was 17 and 9% (Figure 2B). There was no statistical difference between syncope combined with spontaneous Type 1 and syncope combined with non-Type 1 (OR 2.05, 95% CI: 0.96 to 4.40, *P* = 0.06; Heterogeneity: *P* = 0.24, *I*^2^ = 30 %, Figure 3Bb). The result of asymptomatic spontaneous Type 1 compared with asymptomatic non-Type 1 was also negative (OR 1.75, 95% CI: 0.56 to 5.48, *P* = 0.34; Heterogeneity: *P* = 0.80, *I*^2^ = 0 %, Figure 3Bc).

### EPS Groups

All 465 patients with a spontaneous Type 1 ECG pattern or non-Type 1 BrS who had undergone EPS were included in the study. The data came from two articles (Sacher et al., 2013; Tokioka et al., 2014). There were 198 spontaneous Type 1 patients with positive EPS, with an event rate of 19%. There were 101 non-Type 1 patients with positive EPS with an event rate of 9%. In the group of negative EPS, the incidence was 10 and 6%, respectively (Figure 2C). In the group of spontaneous Type 1 with positive EPS and non-Type 1 with positive EPS, the results showed no statistical significance (OR 2.09, 95% CI: 0.95 to 4.62, *P* = 0.07; Heterogeneity: *P* = 0.48, *I*^2^ = 0 %, Figure 3Ca). However, among patients with spontaneous Type 1 ECG, those with a positive EPS vs. negative EPS, were at higher risk (OR 2.35, 95% CI: 1.10 to 5.04, *P* = 0.03; Heterogeneity: *P* = 0.73, *I*^2^ = 0 %, Figure 3Cb). The Funnel plot did not show a major publication bias.

## Discussion

In this study, our principal aim was to pool data from several large trials to systematically evaluate the risk for arrhythmic events associated with a spontaneous Type 1 Brugada ECG when combined with one or more additional risk factors. We included data from 4 large trials comprising a total of 1,338 patients. The principal conclusions are as follows: (i) male patients who developed Type 1 Brugada ECGs spontaneously were at a statistically higher risk for developing arrhythmic events than those with non-Type 1 ECGs, but surprisingly this was not the case for females; (ii) there was no statistical difference in arrhythmic event risk between spontaneous Type 1 and non-Type 1 among patients who had history of syncope; and (iii) patients with a spontaneous Type 1 ECG pattern combined with positive EPS were at higher risk than those with a negative EPS.

### Type 1 and Non-type 1 Groups

Above all, a positive result was obtained and there were significant differences between the two groups: Type 1 and non-Type 1. The prognostic significance of Type 1 in BrS was poor and during the follow up the incidence of arrhythmic events was high. On the whole, most articles were in support of Type 1 as an independent risk factor of BrS. De Asmundis et al. (2017) found that spontaneous Type 1 ECG was independently associated with higher risk of malignant arrhythmias in the follow-up period. They found that spontaneous Type 1 was able to predict arrhythmic events because the sensitivity was 66% and positive predictive value was 52%. And the absence of it was especially helpful to identify patients with a low risk of VF/SCD with high values of specificity of 95% and negative predictive value of 94.3%. Kamakura et al. (2009) reported that the long-term prognosis of patients in Type 1 group and that of non–Type 1 group were similar. Because the annual incidence of arrhythmic events was no difference between the 2 groups (Type 1: 23%, non-Type 1: 14%, *P* = 0.67). Their main finding was that patients with a non-Type 1 ECG, even in the case of a sodium channel blocker challenge, did not necessarily have a better prognosis than those with spontaneous or drug-induced Type 1 ECG. In their prospective study, a spontaneous Type 1 ST-elevation was not a reliable indicator, including only probands.

### Gender Groups

Male patients with spontaneous Type 1 ECG patterns displayed a higher risk profile and event rate when compared to non-Type 1 male patients. In females, the risk of cardiac events did not significantly differ between spontaneous Type 1 and non-Type 1. A total of 1,008 individuals were included. Women with a Type 1 diagnosis (12%) had a lower incidence (8%) of events compared to men (Type 1, 88%; incidence, 16%). Benito et al. (2008b) found that among male patients with spontaneous Type-1 ECG, cardiac events were more frequent. Male patients with cardiac events were more likely to display a spontaneous Type 1 ECG (*p* < 0.05) than male patients who did not develop cardiac events. It is noteworthy that Type 1 ECG at time of diagnosis was significantly more frequent in men than in women (47 vs. 23%, *p* < 0.001). Sacher et al. (2013) came to a conclusion consistent with ours. They showed that in many large studies, spontaneous Type 1 ECG was a risk factor for arrhythmic events; these studies were largely comprised of males. Consistent with our findings, they pointed out that risk factors established from a mostly male population may not help to identify high-risk females. Finally, they concluded that that spontaneous Type 1 ECG and the degree of ST elevation were not associated with more severe symptoms in female BrS patients. Shi et al. (2017) found that in adult Asian males, the frequency of Brugada ECG pattern (Type 1) was especially prevalent. Although our study included large international multicenter cohorts, the number of female patients is still relatively small. This limits the statistical power of the study, but it is noteworthy that this is the largest comparison thus far comparing males and female patients with Type 1 vs. non-Type 1 ECGs. One may realize that there is often a limited systematic reference analysis related to BrS (Martini and Nava, 2009), so the clinical relevance and potential importance of this study is clear.

### SCA, Syncope, and Asymptomatic Groups

Our data suggest that although the risk of spontaneous Type 1 BrS patients with SCA is similar to that of non-Type 1 BrS patients with SCA, we should not underestimate the risk of patients with non-Type 1 Brugada pattern who survived an episode of SCA. Consistent with this conclusion, Sacher et al. (2013) using multivariate analysis reported that the only factor predictive of appropriate device discharge was the presence of preimplantation symptoms such as resuscitated SCA (HR, 10.149; 95% CI, 4.364–23.607).

In patients with a history of syncope, we found no statistically significant difference between BrS patients with and without at Type 1 ECG. Priori et al. (2012) concluded that the presence of a spontaneous Type 1 ECG and history of syncope were the only predictors of adverse outcome. Thus, a risk stratification scheme was proposed that ICD was only recommended for patients with a spontaneous ST-segment elevation combined with syncope (Priori et al., 2012). In contrast, a drug-induced ECG pattern combined with syncope had a very high negative predictive value. It was deemed mandatory to implant a defibrillator in these patients, until other effective treatments could be developed (Milman et al., 2018a). Guidelines (Priori et al., 2013) suggest that ICD may be useful when a spontaneous Type 1 ECG and history of syncope are present. Several studies have shown that syncope is an independent predictor of risk. BrS patients who suffered from previous syncope were more likely to exhibit EPS inducibility (Priori et al., 2013). Many studies provided sufficient evidence, showing that syncope was an independent predictor of risk (Brugada et al., 2004; Priori et al., 2012; Calvo et al., 2016).

### EPS Groups

Finally, our results suggest that patients with spontaneous Type 1 ECG pattern are at increased risk when associated with a positive EPS study as compared with a negative EPS result. To our knowledge, no previous study evaluated the risk of patients displaying a spontaneous Type 1 ECG combined with EPS results. Giustetto et al. (2009) demonstrated the predictive value of a negative EPS. The role of programmed electrical stimulation (PES) in risk stratification of BrS has remained a topic of debate (Priori et al., 2012). Delise et al. (2011) proposed a multi parameter (clinical combined with electrophysiological) method to help identify high-risk groups. They concluded that electrophysiologic study can be helpful when evaluated with other clinical risk factors. A positive EPS combined with other risk factors contribute to the decision of ICD implantation. And the high-risk populations are those with spontaneous Type 1 ECG and at least two risk factors (Delise et al., 2011). A study involving a pooled analysis showed that an inducibility of VF could identify BrS patients with increased risk for cardiac arrest and was a significant predictor of VF events, when VT/VF was induced with single or double extra-stimuli (Sroubek et al., 2016). Recently, Pappone et al. (2018) showed that among BrS patients the extent of substrate was the only independent predictor of inducibility of VT/VF and could be used as a new marker for risk stratification and treatment. The most recent HRS/APHRS/EHRA/SOLAECE expert consensus report recommends that EPS may be considered in asymptomatic individuals with spontaneous Type 1 Brugada pattern and that if VT/VF is inducible with ≤ 2 extra-stimuli, an ICD should be considered (Antzelevitch et al., 2016). In asymptomatic patients with a spontaneous Type 1 Brugada ECG pattern, further risk stratification can be achieved by an EPS with PVS using single and double extra-stimuli (Al-Khatib et al., 2017).

## Conclusions

In summary, our pooled analysis first indicates that spontaneous Type 1 ECG patterns display a higher risk profile than non-Type 1 in the male population, but not in women. The case of spontaneous Type 1 ECG patterns combined with being male may be an independent risk factor. And patients with a non-Type 1 Brugada pattern who survived an episode of SCA should not be underestimated with regards to the risk for arrhythmic events. The prognosis of spontaneous Type 1 BrS patients is worse when combined with positive results of EPS. These findings will provide further evidence for detailed risk stratification to guiding clinical practice and preventing sudden cardiac death.

## Limitations

Despite the collection of pooled data, the number of females with BrS in our study is relatively small, limiting the statistical power of the study. The influence of sex, history of syncope, and EPS on spontaneous Type 1 BrS will need to be confirmed in larger populations of BrS patients.

## Author's Note

All authors take responsibility for all aspects of the reliability and freedom from bias of the data presented and their discussed interpretation.

## Author Contributions

YX, XL, and DH designed the study. FS and KK contributed to the original data. XL, HB-M, FS, KK, NL, YL, YG, TL, HS, and DH performed the study of clinical subjects. DH, CA, XL, and YX coordinated the clinical evaluations and summarized the database. DH, XL, and YX developed the conceptual approaches to data analysis. XL, DH, YX, CA, and HB-M wrote the manuscript. All coauthors contributed to editing of manuscript.

### Conflict of Interest Statement

The authors declare that the research was conducted in the absence of any commercial or financial relationships that could be construed as a potential conflict of interest.
