# Supplementary material for: Pooled Analysis of Risk Stratification of Spontaneous Type 1 Brugada ECG: Focus on the Influence of Gender and EPS
Source: Front Physiol. 2019 Jan 31;9:1951. doi: 10.3389/fphys.2018.01951 (PMC6365464; doi:10.3389/fphys.2018.01951)
Supplement: Supplementary file 1 [file Data_Sheet_1.docx]

**Online Supplement**

**Pooled Analysis of** [**Risk** **Stratification**](https://www.ncbi.nlm.nih.gov/pubmed/28994463) **of**

**Spontaneous Type 1 Brugada ECG: Focus on the Influence of Gender and EPS**

*Running head: Pool study of Spontaneous Type 1 BrS*

**Figure Legend**

Supplemental Figure 1. Forest plots comparing outcomes of Type 1 BrS and non-Type 1 BrS.


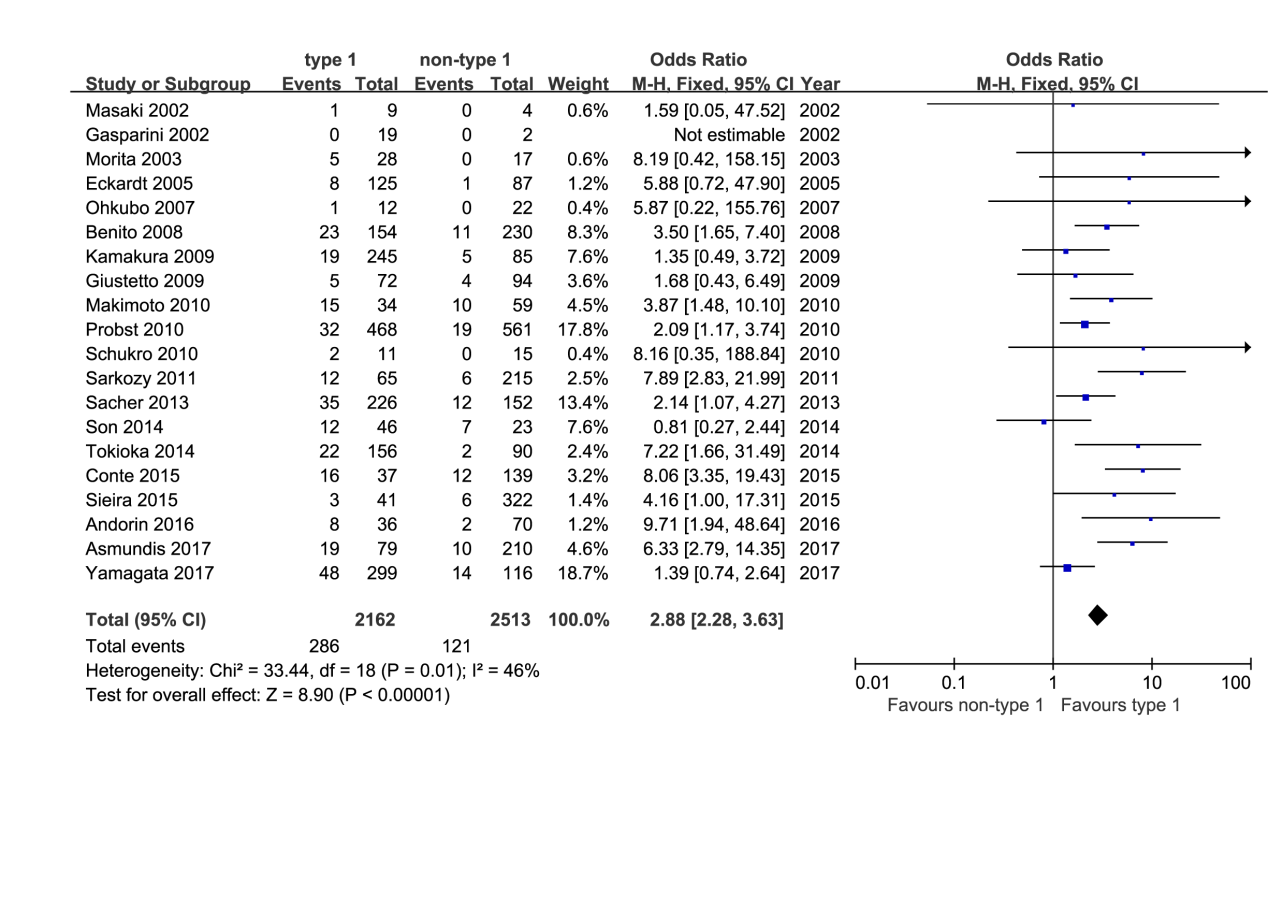


Supplemental Figure 1.

**Supplemental Table 1. Study characteristics of 20 studies included in pooled analysis**

| Investigator | Location | Type of study | Study of design | Study population | Mean follow-up | Endpoint | Quality score |
| --- | --- | --- | --- | --- | --- | --- | --- |
| Masaki et al.,2002 | Japan | SC | PS | Patients with the RBBB-STE ECG ,with an ECG pattern consisting of right bundle branch block with ST elevation in leads V1 to V3 | 34±32 months | SPVT/VF | 15 |
| Gasparini et al.,2002 | Italy | SC | PS | Patients with Brugada syndrome underwent a PES protocol from two right ventricular sites | 42 months | PES protocol completion/Induction of sustained/Reproducible( > 6 consecutive inductions) nonsustained ( > 6 beats) fast ventricular arrhythmia | 15 |
| Morita et al.,2003 | Japan | SC | PS | Patients with Brugada-type ECG | 38±27 months | PVT/VF | 16 |
| Eckardt et al.,2005 | Europe | MC | RS | Patients with a Type 1 Brugada ECG pattern | 40±50 months | Arrhythmic event（ICD intervention as a result of a SVT/SVF）/SCD | 16 |
| Ohkubo et al.,2007 | Japan | SC | PS | Patients with a Brugada-type ECG | 47.1±33.7 months | Sudden cardiac death/VF | 14 |
| Benito et al.,2008 | Canada | MC | PS | Patients with spontaneous or induced (after sodium-blocker admin-istration) coved-type ECG pattern (type-1 ECG) | 58±48 months | SCD/Documented ventricular fibrillation | 15 |
| Giustetto et al.,2009 | Italy | SC | PS | Patients with a brugada Type1 ECG ,spontaneously or after pharmacological testing with Class 1 C drugs | 30±21 months | VF/VT/Sudden death | 16 |
| Kamakura et al.,2009 | Japan | MC | RS | Patients with a Type 1 or non-Type 1 Brugada ECG pattern | 48.7±15.0 months | VF/Sudden death | 18 |
| Makimoto et al.,2010 | Japan | MC | RS | Patients with BrS | 75.7±38.4 months | SCD/VF/Sustained VT | 20 |
| Probst et al.,2010 | Europe | MC | RS | Patients with Type 1 ECG present either at baseline or after drug challenge | 31.9 months | Cardiac events (Appropriate ICD shocks/SCD) | 17 |
| Schukro et al.,2010 | Austria | MC | RS | Patients with a Brugada type ECG pattern | 57.0± 32.2 months | Arrhythmic event | 14 |
| Sarkozy et al.,2011 | Barcelona | SC | PS | Patients with diagnostic type I Brugada ECG pattern | 59 months | VF/SD | 15 |
| Sacher et al.,2013 | France | MC | PS | Patients with a Type 1 Brugada ECG pattern implanted with an ICD | 77±42 months | Death/Inappropriate shock | 16 |
| Tokioka et al.,2014 | Japan | SC | RS | Patients with a Brugada-type ECG | 45.1 months | VF/SCD | 17 |
| Son et al.,2014 | Korea | SC | RS | Patients with spontaneous Type 1 ECG implanted with ICD | 59±46 months | Appropriate shock/Inappropriate shock/Cardiac causes | 15 |
| Sieira et al.,2015 | Belgium | SC | RS | Patients with spontaneous or drug-induced Brugada Type I ECG and no symptoms | 73.2±58.9 months | ICD shock/SCD/Aborted SCD | 18 |
| Conte et al.,2015 | America | SC | RS | Patients with spontaneous or drug-induced Brugada Type 1 ECG and ICD implantation and continuous follow-up at a single institution | 83.8±57.3 months | Spontaneous sustained Vas/Death/ICD shocks/Inappropriate shocks/Device-related complications | 16 |
| Andorin et al.,2016 | Europe | MC | RS | Patients with diagnosis of BrS，spontaneous or drug-induced | 54 months | Sudden death/VT/VF/Appropriate ICD shock | 15 |
| Yamagata et al.,2017 | Japan | MC | RS | Patients with Brugada syndrome whose SCN5A gene was analyzed for mutations | 72 months | Earliest appropriate ICD shock / Aborted cardiac arrest/SCD | 16 |
| Asmundis et al.,2017 | America | SC | RS | Patients with a Brugada Type 1 ECG | 120.6±55.7 months | VF/SCD | 16 |

BrS, Brugada syndrome; ECG, electrocardiogram; ICD, implantable cardioverter defibrillator; MC, multicenter study; MINOR, methodological index for non-randomized studies; NA, not available; PS, prospective study; RS, retrospective study; SC, single center study; SCD, sudden cardiac death; VF, ventricular fibrillation; VT, ventricular tachycardia.

**Supplemental Table 2-1. Clinical characteristics of study patients.**

|  | Masaki  et al.,2002 | | Gasparini et al.,2002 | Ohkubo et al.,2007 | Benito et al.,2008 | Giustetto et al.,2009 | Schukro et al.,2010 | Sarkozy et al.,2011 | Sacher et al.,2013 | Tokioka et al.,2014 | Yamagata  et al.,2017 |
| --- | --- | --- | --- | --- | --- | --- | --- | --- | --- | --- | --- |
| Total patients, n | | 13 | 21 | 34 | 384 | 166 | 26 | 280 | 378 | 246 | 415 |
| Male/female, n | | 12/1 | 18/3 | 33/1 | 272/112 | 138/28 | 20/6 | 168/40 | 310/68 | 236/10 | 403/12 |
| Age (years) | | 52.4±11.0 | 34±15 | 52±13 | 48±18 | 45±14 | 43.2±11.6 | 41±18 | 46±13 | 47.6±13.6 | 46±14 |
| Spontaneous  Brugada ECG, n (%) | | 9(69) | 19(90) | 12(35) | 154(40) | 72(43) | 11(42) | 65(23) | 226(60) | 156(63) | 299(72) |
| Non-spontaneous type1 Brugada ECG, n (%) | | 4(31) | 2(10) | 22(65) | 230(60) | 94(57) | 15(58) | 215(77) | 152(40) | 90(37) | 116(28) |
| Family history of  SCD, n (%) | | NA | 8(38) | 3(9) | NA | 39(23) | NA | 149(53) | 111(29) | 69(28) | 64(15) |
| History of syncope, n (%) | | 3(23) | 8(38) | 9(26) | 65(17) | NA | 7(27) | NA | 181(48) | 40(16) | 10(2) |
| Type1 Syncope  total, n (%) | | NA | NA | NA | NA | NA | NA | NA | 107(28) | 28(11) | NA |
| Type1 Syncope  events, n (%) | | NA | NA | NA | NA | NA | NA | NA | 18(5) | 12(5) | NA |
| Asymptomatic  Type1 total, n (%) | | 5(38) | 12(57) | 23(68) | 103(27) | NA | 15(58) | NA | 166(44) | NA | NA |
| SCN5A mutation,  n (%) | | NA | 8(38) | NA | 95(25) | NA | NA | NA | 41(11) | 17(7) | 60(14) |
| EPS | |  |  |  |  |  |  |  |  |  |  |
| Stimulation sites | | RVA+  RVOT | RVA+RVOT | RVA+  RVOT | RVA | RVA+RVOT | RVA+  RVOT | NA | NA | RVA+  RVOT+  LV | RVA+RVOT |
| Extra stimuli | | Up to 3 | Up to 3 | NA | Up to 3 | Up to 2 | Up to 3 | NA | Up to 3 | Up to 3 | Up to 3 |
| Basic cycle lengths | | 2 cycles | 600/500/  400 ms | 2 cycles  (600 and 400 ms) | 600, 500, and 430 ms | 600 and 400 ms | 500, 430, 375, and 333 ms | NA | NA | 2 cycles | >200 ms |
| Patients with PVS, n(%) | | 13(100) | 21(100) | 34(100) | 350(91) | 135(81) | 14(54) | 238(85) | 311(82) | 155(63) | 339(82) |
| Inducible VT/VF, n(%) | | 8(62) | 14(67) | 27(79) | 95(27) | 46(34) | 2(14) | 61(26) | 228(73) | 71(46) | 191(56) |
| fQRS (+), n (%) | | NA | NA | NA | NA | NA | NA | NA | NA | 78(32) | NA |

**Supplemental Table 2-2. Clinical characteristics of study patients.**

|  | Morita et al.,2003 | Eckardt et al.,2005 | Kamakura  et al.,2009 | Makimoto et al.,2010 | Probst et al.,2010 | Sieira  et al.,2015 | Andorin et al.,2016 | Son  et al.,2014 | Conte  et al.,2015 | Asmundis et al.,2017 |
| --- | --- | --- | --- | --- | --- | --- | --- | --- | --- | --- |
| Total patients, n | 45 | 212 | 330 | 93 | 1029 | 363 | 106 | 69 | 176 | 289 |
| Male/female, n | 45/0 | 152/60 | 315/15 | 91/2 | 745/284 | 200/163 | 58/48 | 68/1 | 118/58 | 203/86 |
| Age (years) | 46±10 | 45±6 | 51.4±14.8 | 46±14 | 45 | 40.9±17.2 | 11.1±5.7 | 46.2±13.5 | 43.3±16.8 | 45±16 |
| Spontaneous  Brugada ECG, n (%) | 9(20) | 125(59) | 173(52) | 34(37) | 468 (45) | 41(11) | 36(34) | 46(67) | 37(21) | 79(27) |
| Non-spontaneous type1 Brugada ECG, n (%) | NA | 87(41) | 157(48) | 59(63) | 561(55) | 322(89) | 70(66) | 23(33) | 139(79) | 210(73) |
| Drug-induced  Brugada ECG, n (%) | 36(80) | 87(41) | 72(22) | NA | 561(55) | 288(79) | 70(66) | NA | NA | NA |
| Family history of SCD,  n (%) | 8(18) | 60(28) | 30(9) | NA | 264(26) | 182(50) | 46(43) | 13(19) | 90(51) | 99(34) |
| History of syncope,  n (%) | 2(4) | 65(31) | 67(20) | 35(38) | 309(30) | NA | 46(43) | NA | 105(60) | 103(36) |
| Asymptomatic, n (%) | 36(80) | 123(58) | 207(63) | 36(39) | 654(64) | NA | NA | 14(20) | 46(25) | 169(58) |
| SCN5A mutation,  n (%) | NA | 32(15) | NA | NA | 650(63) | NA | 58(55) | NA | 23(22) | 51(18) |
| EPS |  |  |  |  |  |  |  |  |  |  |
| Stimulation sites | RVA+Septal RVOT+FW RVOT  LV | RVA+  RVOT | RVA+  RVOT | RVA+  RVOT | NA | RVA | NA | NA | NA | NA |
| Extra stimuli | Up to 3 | Up to 3 | Up to 3 | Up to 3 | Up to 3 | NA | Up to 3 | Up to 3 | Up to 3 | NA |
| Basic cycle lengths | NA | 500/430/  370/330ms | NA | NA | NA | 600,500, and 430ms | NA | NA | >200 ms | NA |
| Patients with PVS, n (%) | 45(100) | 212(100) | 232(70) | 78(84) | 638(62) | 321(88) | 22(21) | 69(100) | 165(94) | 280(97) |
| Inducible VT/VF, n (%) | 34(76) | 168(79) | 22(9) | 59(76) | 262(41) | 32(10) | 9(41) | 14(20) | 72(44) | 52(19) |
| fQRS (+), n (%) | NA | NA | NA | NA | NA | NA | NA | NA | 1(0) | 50(17) |

ECG, electrocardiogram; EPS, electrophysiological study; fQRS, fragmented QRS; FW RVOT, free-wall region of the ROVT; LV, left ventricle; NA, not available; n, number; RVA, right ventricular apex; RVOT, right ventricular outflow tract; SCD, sudden cardiac death; VF, ventricular fibrillation; VT, ventricular tachycardia.
